# Effect of episomally encoded DNA polymerases on chemically induced mutagenesis at the *hisG46* target in Ames test

**DOI:** 10.1186/s41021-020-00154-2

**Published:** 2020-03-24

**Authors:** Petr Grúz, Masatomi Shimizu, Kei-ichi Sugiyama, Masami Yamada, Masamitsu Honma

**Affiliations:** 1 Division of Genetics and Mutagenesis, National Institute of Health Sciences, 3-25-26 Tonomachi, Kawasaki-ku, Kawasaki-shi, Kanagawa 210-9501 Japan; 2 Division of Medical Nutrition, Faculty of Healthcare, Tokyo Healthcare University, Tokyo, 154-8568 Japan; 3 Department of Applied Chemistry, National Defense Academy, 1-10-20 Hashirimizu, Yokosuka, Kanagawa 239-8686 Japan

**Keywords:** Ames test, DNA polymerase, samAB, Mutagenesis, Azide, pKM101

## Abstract

**Background:**

The standard Ames test strains owe their high sensitivity to chemical and physical mutagens to the episomal Y-family DNA polymerase RI encoded by the *mucAB* operon. The *S. typhimurium* test strains carry also another related *samAB* operon on a 60-kDa cryptic plasmid. In contrast to the chromosomally encoded Y-family DNA polymerases V and IV, these plasmid born polymerase genes have no direct counterpart in mammalian cells. By replicating damaged templates, DNA polymerases play a central role in mutagenesis and genome stability. It is therefore imperative to investigate their specificity to understand differences in mutagenesis between the prokaryotic versus eukaryotic (mammalian) systems. To this end we have isolated and separately expressed the DNA polymerase subunits encoded by the *mucAB* and *samAB* operons. After demonstrating how these enzymes control chemical and UV mutagenesis at the standard *hisD3052* and *hisG428* Ames test targets, we are now adding the third Ames test target *hisG46* to the trilogy.

**Results:**

Four new Ames tester strains based on the *hisG46* target have been constructed expressing the activated DNA polymerase MucA’ and SamA’ accessory subunits combined with the MucB and SamB catalytical subunits under the control of *lac* promoter. These polymerase assemblies were substituted for the endogenous PolRI, PolV and SamAB polymerases present in the standard TA100 strain and tested for their abilities to promote chemically induced mutagenesis. SamA’ + SamB has been able to promote mutagenesis induced by AF-2 and 1,8-DNP to higher extent than SamA’ + MucB. The MucA’ + MucB (PolRI*) more efficiently promoted MMS as well as spontaneous mutagenesis than its wild type counterpart but was less efficient for other mutagens including AFB1. Strikingly azide mutagenesis was inhibited by PolRI and also SamA’B.

**Conclusion:**

A new system for SOS-independent overexpression of the activated DNA polymerases RI and SamA’B and their chimeras in the *hisG46* Ames test background has been established and validated with several representative mutagens. Overall, the TA100 strain showed the highest sensitivity towards most tested mutagens. The observed inhibition of azide mutagenesis by PolRI* suggests that this type of Y-family DNA polymerases can perform also “corrective” *error free* replication on a damaged DNA.

## Introduction

The Ames test has been considered a golden standard for the mutagenicity assays and is used worldwide by the government regulatory agencies to assess the safety of chemicals [1]. DNA polymerases play a key role in mutagenesis by deciding whether unrepaired damaged DNA will be tolerated and replicated often at the expense of altering the genetic code and hence introducing mutations or whether it will arrest cell division ultimately leading to death. They also shape the mutational specificity of DNA damaging genotoxicants. The Y-family DNA polymerases are evolutionary preserved group of enzymes specializing in “recognizing” and bypassing certain types of endogenous DNA lesions in an *error-free* manner but are highly *error-prone* on intact DNA or when replicating through other types of DNA lesions than they have evolved to recognize [2].

In bacteria, many Y-family DNA polymerases are encoded by plasmids spreading antibiotic resistances and have possible role in bacterial evolution helping to adapt to changing environments [3, 4]. One of the highest *error prone* DNA polymerases is DNA polymerase RI encoded by the *mucAB* operon carried on the plasmid pKM101 [5, 6]. It is utilized in the Ames test where the pKM101 plasmid incorporated in the standard battery of strains greatly enhances sensitivity to various chemical and physical mutagens [7]. Additionally, all of the *S. typhimurium* Ames tester strains carry another 60-MDa cryptic plasmid pYQ100 which encodes the *samAB* operon, homologous to *mucAB,* that is inefficient in promoting mutagenesis at its natural expression levels [4, 8]. A third Y-family DNA polymerase encoded by the chromosomal *umuDC*_ST_ operon is also present in all of the *S. typhimurium* Ames tester strains [9, 10]. These strains possess yet fourth DNA polymerase encoded by the *dinB*_ST_ gene located also on the chromosome that is not contributing in greater extent to response to most mutagens similarly to *samAB* and *umuDC*_ST_ [11].

The activity of the Y-family polymerase core catalytical subunit such as UmuC is controlled by the accessory subunit UmuD that has to be proteolytically activated to its shorter form designated UmuD’ by removal of N-terminal tail to trigger the translesion DNA synthesis (TLS) [12]. An exception is the bacteriophage P1 that encodes already activated i.e. shorter version of UmuD called HumD [13]. This mechanism seems common to all the Y-family polymerases whose catalytic subunits are encoded by the second gene in an operon like *mucAB*, *umuDC* and *samAB* but not *dinB*. In this case the whole biochemically active Y-family DNA polymerase holoenzyme assembly requires also other factors like the RecA and Ssb proteins [6] and may also integrate some additional accessory proteins encoded by adjacent genes carried on conjugative plasmids such as in the case of *impCAB* operon [4]. Although DNA polymerase IV encoded by the *dinB* gene can replicate DNA on its own in a test tube, association with the β-subunit sliding clamp tethering it to DNA is required for achieving reasonable processivity [14–16] and this is also true for the all other DNA polymerases.

Since the Ames test is used to judge the mutagenicity of chemicals in humans, which greatly differ in the DNA polymerase repertoire, it is important to achieve the same mutation specificity in the enterobacterial cells as in the mammalian system. It is therefore important to study the properties and specificity of the prokaryotic DNA polymerases to estimate to which extent we can extrapolate the results from the Ames test to the human population. The TLS DNA polymerases play a key role in the process of converting DNA lesions to specific mutations. Although the chromosomally encoded DNA polymerases IV (*dinB*) and V (*umuDC*) are the orthologs of the human DNA polymerases κ and η, respectively [17], the episomal DNA polymerases RI and SamAB of *S. typhimurium* are unique to bacteria and even cause malignant transformation when introduced into mouse cells [18]. It is therefore important to scrutinize the mechanisms by which the episomally encoded DNA polymerases promote mutagenesis especially in the view that the response to most mutagens in the Ames test depends on the action of the plasmid-born DNA polymerase RI which is a very powerful mutator. We have previously split the *mucAB* and *samAB* operons and separately expressed the activated polymerase subunits from the IPTG-controllable *lac* promoter allowing to test them at various combinations, expression levels and in response to chemical or physical mutagens. To examine different mutation specificity we have already analyzed the polymerase abilities to promote base substitutions at the AT base pair (*hisG428* [19];) as well as − 2 GC frameshift (*hisD3052* [20];) targets of the standard Ames tester strains previously. To complete the “trilogy” we are now presenting the response of the third major Ames test target - the GC base pair substitution hotspot at the *hisG46* locus - to 5 representative mutagens in the presence of various combinations of the episomal DNA polymerases.

## Materials and methods

### Chemicals

The sources of chemicals used in this study were as follows: Furylfuramide (AF-2; CAS No. 3688-53-7) and isopropyl β-D-thiogalactopyranoside (IPTG) were from Wako Pure Chemical Industries, Osaka, Japan; Methylmethane sulfonate (MMS; CAS No. 66–27-3) was purchased from Aldrich Chemical Co., Milwakukee, WI; AFB1 (CAS No. 1162–65-8) came from Sigma Chemicals, St. Louis, MO; 1,8-dinitropyrene (1,8-DNP; CAS No. 42397–65-9) was a gift from Dr. Naoki Miyata, National Institute of Health Sciences, Tokyo, Japan and azidoglycerol (AG; CAS No. 73018–99-2) synthesized as described previously [21] was kindly provided by Dr. T. Gichner, Institute of Experimental Botany, Academy of Sciences of the Czech Republic, Prague, Czech Republic. The test mutagens were dissolved in DMSO prior to use except MMS and AG which were dissolved in water. Rat liver 9000 x g supernatant fraction (S9) from animals pretreated with phenobarbital and 5,6-benzoflavone was purchased from Kikkoman, Chiba, Japan. All other chemicals were of analytical purity and have been purchased from local supplier (Wako Pure Chemical Industries, Osaka, Japan).

### Bacterial strains and plasmids

The strains used in this study are all derived from the standard Ames tester strain TA1535 carrying the *hisG46* base substitution mutation target [1, 22]. The standard TA1535 and TA100 strains had been originally obtained from *Bruce Ames,* University of California, Berkeley. The strain YG5151 has been constructed by the preligation method routinely used for the disruption of *Salmonella umuDC*_*ST*_ and *samAB* genes in our laboratory utilizing the kanamycin and chloramphenicol resistance cassettes as described previously [8, 20, 23] and became a base for the construction of other strains used throughout this study. To create the PolV-deficient variant of strain TA100 named YG9056, the plasmid pKM101 has been introduced into the strain YG5151 by the bacterial conjugation technique using the *E.coli* strain WP2 as a donor. Other strains expressing the active forms of the episomal PolRI and SamAB DNA polymerases from an IPTG-inducible *lac* promoter, i.e. YG9028 and YG9029, as well as their chimerical cross-variants, i.e. YG9032 and YG9033, have been constructed by sequential transformation with the plasmids pYG8524 or pYG8529 expressing the *lacI*^*q*^ repressor followed by pYG8517 or pYG8518. The transformation with first passing the plasmids through the methylation helper *S. typhimurium* strain LB5000 has been carried out essentially the same as described for the related strains carrying the *hisG428* (same as e.g. in TA104) and *hisD3052* (same as e.g. in TA98) targets constructed in our laboratory previously [19, 20].

### Mutagenicity assay

**Fig. 1:**
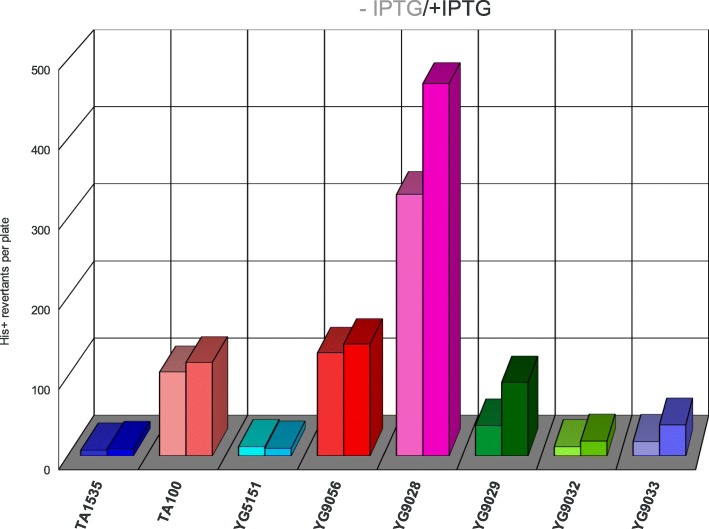
Comparison of spontaneous revertant counts in the newly developed Ames tester strains. The bar graph demonstrates levels of spontaneous mutagenesis in the classical Ames tester strains (TA1535, TA100) and their PolV-deficient derivatives (YG5151, YG9056) compared to the newly constructed strains overexpressing the activated forms of PolRI* (MucA’ + MucB, YG9028), the SamA’B polymerase (SamA’ + SamB, YG9029) and their chimerical combinations (MucA’ + SamB, YG9032; SamA’ + MucB, YG9033). The darker bars on the right side of the data point pairs represent the numbers of revertants at higher DNA polymerase expression levels induced by IPTG

**Fig. 2:**
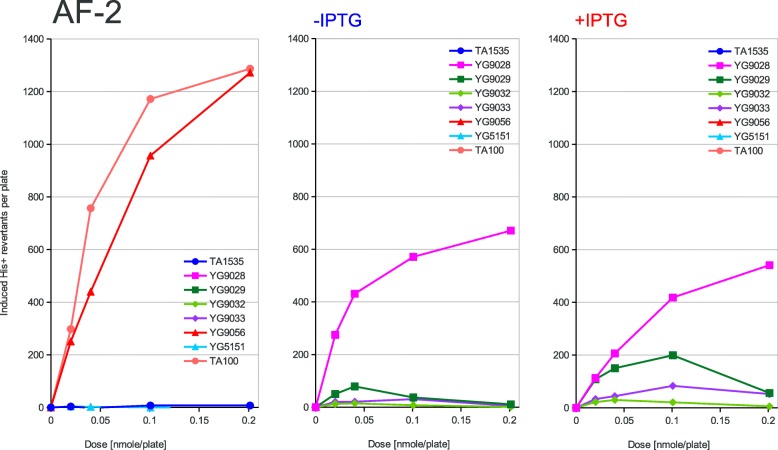
Dose dependence of mutagenesis induced by AF-2**.** Furylfuramide (AF-2) has been applied at the doses indicated on the X-axis to the classical Ames tester strains and their PolV-deficient derivatives (left panel) or to the new Ames tester strains overexpressing the activated PolRI* and SamA’B polymerase subunits from the uninduced (middle panel) or induced (right panel) *lac* promoter. The induced His+ revertant counts displayed on the Y-axis were calculated by subtracting the average spontaneous revertant number for a given strain from the total number of revertants per plate values. The strains used are shown in the graph legend and described in Table 1 in detail

**Fig. 3:**
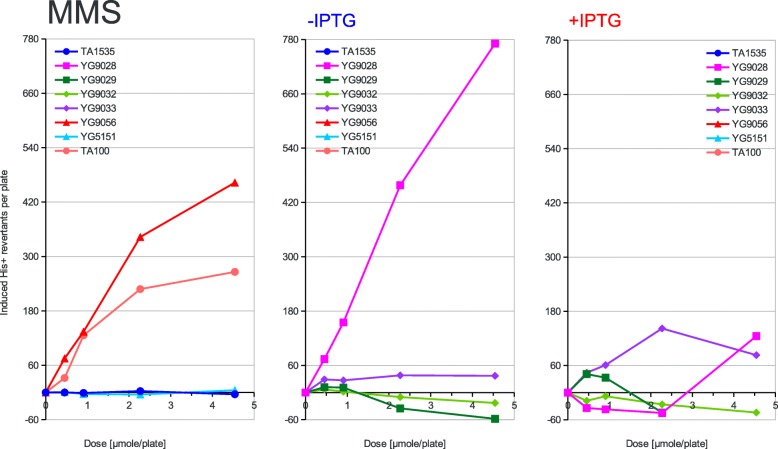
Dose dependence of mutagenesis induced by MMS. Methyl methanesulfonate (MMS) has been applied at the doses indicated on the X-axis to the classical Ames tester strains and their PolV-deficient derivatives (left panel) or to the new Ames tester strains overexpressing the activated PolRI* and SamA’B polymerase subunits from the uninduced (middle panel) or induced (right panel) *lac* promoter. The induced His+ revertant counts displayed on the Y-axis were calculated by subtracting the average spontaneous revertant number for a given strain from the total number of revertants per plate values. The strains used are shown in the graph legend and described in Table 1 in detail

**Fig. 4:**
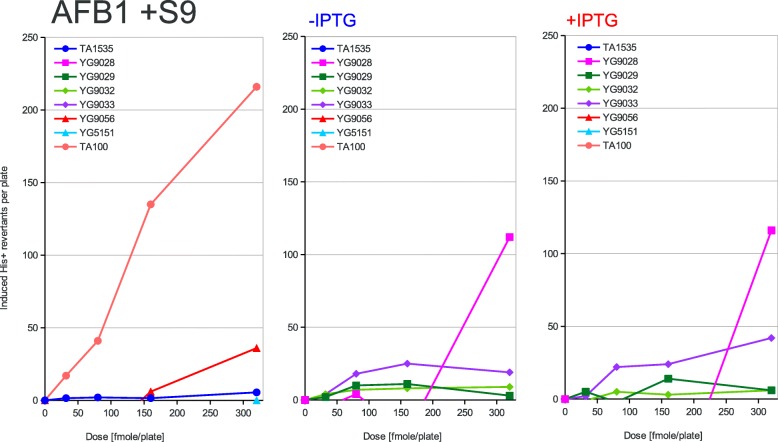
Dose dependence of mutagenesis induced by AFB1. Aflatoxin B1 (AFB1) has been applied at the doses indicated on the X-axis to the classical Ames tester strains and their PolV-deficient derivatives (left panel) or to the new Ames tester strains overexpressing the activated PolRI* and SamA’B polymerase subunits from the uninduced (middle panel) or induced (right panel) *lac* promoter. The induced His+ revertant counts displayed on the Y-axis were calculated by subtracting the average spontaneous revertant number for a given strain from the total number of revertants per plate values. The strains used are shown in the graph legend and described in Table 1 in detail. To activate the mutagen, 20 min preincubation with rat liver S9 fraction has been carried out before plating as described in the Materials and methods section

**Fig. 5:**
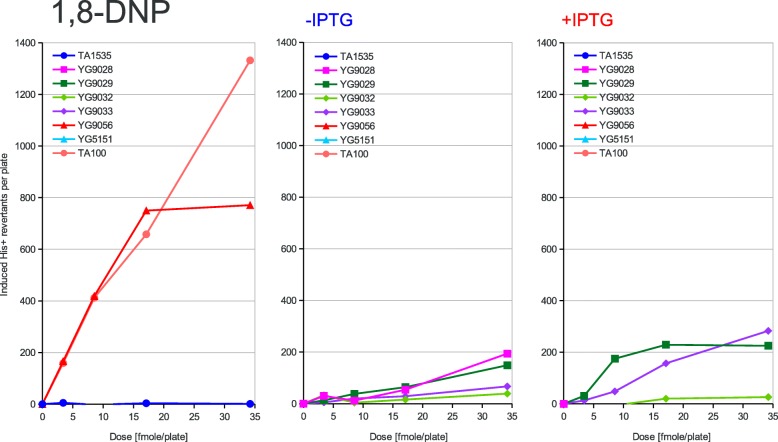
Dose dependence of mutagenesis induced by 1,8-DNP. 1,8-Dinitropyrene (1,8-DNP) has been applied at the doses indicated on the X-axis to the classical Ames tester strains and their PolV-deficient derivatives (left panel) or to the new Ames tester strains overexpressing the activated PolRI* and SamA’B polymerase subunits from the uninduced (middle panel) or induced (right panel) *lac* promoter. The induced His+ revertant counts displayed on the Y-axis were calculated by subtracting the average spontaneous revertant number for a given strain from the total number of revertants per plate values. The strains used are shown in the graph legend and described in Table 1 in detail

**Fig. 6:**
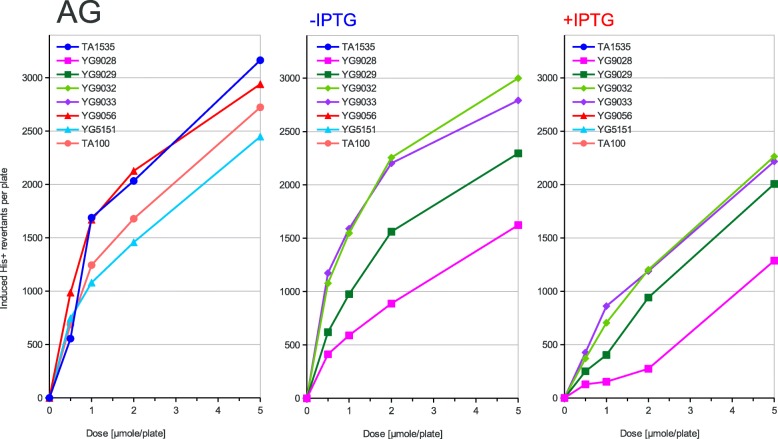
Dose dependence of mutagenesis induced by AG. Azidoglycerol (AG) has been applied at the doses indicated on the X-axis to the classical Ames tester strains and their PolV-deficient derivatives (left panel) or to the new Ames tester strains overexpressing the activated PolRI* and SamA’B polymerase subunits from the uninduced (middle panel) or induced (right panel) *lac* promoter. The induced His+ revertant counts displayed on the Y-axis were calculated by subtracting the average spontaneous revertant number for a given strain from the total number of revertants per plate values. The strains used are shown in the graph legend and described in Table 1 in detail

The standard Ames mutagenesis assay has been carried out as described previously with the optional 20-min preincubation procedure using S9 in the case of AFB1 [20]. The overnight cultures were grown in Bacto Nutrient Broth supplemented with 0.2% glucose and the following antibiotics as required: kanamycin at 10 μg/ml + chloramphenicol at 5 μg/ml (for all YG5151 based strains), ampicillin at 25 μg/ml (for the strains harboring plasmid pKM101) or ampicillin at 50 μg/ml (for the strains harboring plasmids pYG8524 or pYG8529) and tetracycline at 10 μg/ml (for the strains harboring plasmids pYG8517 or pYG8518). The S9 microsomal homogenate has been diluted 10x in 0.1 M phosphate buffer with the cofactors (G-6-P 5 mM, NADPH + NADH 4 mM, MgCl_2_ 8 mM, KCl 33 mM) and applied at an equivalent of 50 μl per plate during the pre-incubation phase used for AFB1. IPTG has been added prior to plating to the top agar at 0.2 mM concentration where indicated to derepress the *lac* promoter. The experiments were repeated at least twice and two plates were used for each dose. The mean values of His^+^ revertants per plate are indicated in Figs. 1, 2, 3, 4, 5 and 6 and the characteristics of used strains are shown in Table 1. Selected data have been subjected to linear regression analysis to calculate the slopes of dose-response curves for each chemical which are the mutagenic potencies expressed as the specific mutagenicity values in Table 2. Only the zero and 3 initial doses within the linear range have been used for this type of analysis unless indicated otherwise as recommended by Bernstein et al. [24].

**Table 1:** Characteristics of *S. typhimurium* strains and plasmids used in the study. Plasmids expressing Y-family DNA polymerase catalytical and accessory subunits incorporated in the Ames test strains used in present study are shown in the lower part

Strain or plasmid	Description	Source
Strains		
TA1535	*his*G46, *gal*, Δ (*chl*, *uvr*B, *bio*), *rfa*	[1]
TA100	As TA1535, but harbors pKM101; Ap^r^	[1]
YG5151	As TA1535, but Δ*umu*DC_ST_::Km^r^, Δ*sam*AB::Cm^r^	This study, [23]
YG9056	As YG5151, but harbors pKM101; + Ap^r^	This study
YG9028	As YG5151, but harbors pYG8524 + pYG8517; + Ap^r^ + Tc^r^	This study, [23]
YG9029	As YG5151, but harbors pYG8529 + pYG8518; + Ap^r^ + Tc^r^	This study
YG9032	As YG5151, but harbors pYG8524 + pYG8518; + Ap^r^ + Tc^r^	This study
YG9033	As YG5151, but harbors pYG8529 + pYG8517; + Ap^r^ + Tc^r^	This study
Plasmids		
pKM101	R46 derivative carrying the *muc*AB operon of *Shigella sonnei*	[7]
pYG8524	pBR322 derivative expressing the activated MucA’ subunit of PolRI from the *lac* promoter	[19]
pYG8529	pBR322 derivative expressing the activated SamA’ polymerase subunit from the *lac* promoter	[19]
pYG8517	pSC101 derivative expressing the MucB catalytic subunit of PolRI from the *lac* promoter	[19]
pYG8518	pSC101 derivative expressing the SamB polymerase subunit from the *lac* promoter	[19]

*Km*^*r*^ resistant to kanamycin, *Cm*^*r*^ resistant to chloramphenicol, *Tc*^*r*^ resistant to tetracycline, *Ap*^*r*^ resistant to ampicillin

**Table 2:** Mutagenic potencies of tested compounds in standard TA1535 and TA100 versus new Ames tester strains overexpressing the episomal Y-family DNA polymerases. Data from tests conducted in the presence of the *lac* promoter inducer as described under Material and Methods are added for comparison in the columns under the IPTG label. The *lac* promoter drives the expression of the activated forms of DNA polymerases RI* and SamA’B overexpressed in the strains YG9028 and YG9029, respectively

Compound	Specific mutagenicity [revertants/plate/nmole]							
	TA1535		TA100		YG9028		YG9029	
		IPTG		IPTG		IPTG		IPTG
AF-2 ^*)^	–	–	18,788	16,381	10,697	5113	1961	3723
MMS	*–*	–	0.103	0.099	0.205	–	–	–
AFB1	–	–	846	692	–	–	–	–
1,8-DNP	–	–	38,465	53,033	2595	neg.	3782	14,195
AG	1.047	1.151	0.817	0.646	0.42	0.128	0.753	0.464

^*)^ only zero plus 2 first doses used for the calculations due to nonlinearity at higher doses

## Results

### Portable polymerase expression system

We have previously constructed a versatile plasmid-based system for the controllable expression of the activated Y-family DNA polymerases encoded by the episomal *muc*A + *muc*B and *sam*A + *sam*B genes in the standard *S. typhimurium* Ames tester strains [19]. Since the A subunits in their shorter active form designated A’ are expressed from an ampicillin resistant pBR322-based middle copy number replicon and the main catalytic subunits designated B are expressed from a compatible low copy number pSC101-based replicon, we could generate 4 types of strains expressing the natural as well as chimeric polymerase subunit combinations, i.e. MucA’ + MucB (YG9028), SamA’ + SamB (YG9029), MucA’ + SamB (YG9032) and SamA’ + MucB (YG9033) as shown in Table 1. The strain YG9028 expresses the active form of the DNA polymerase RI, sometimes referred to as PolRI*, which has been already biochemically characterized in our and other labs and which is the enzyme responsible for the great sensitivity of the Ames tester strains like TA100, TA104, TA98 or TA97 to various chemical and physical mutagens [6, 25]. In order to investigate the effect of these TLS DNA polymerases on mutagenesis in a “clean background” we have removed the other Y-family DNA polymerase PolV (encoded by the *umuDC*_ST_ operon) from the chromosome as well as deleted the copy of *samAB* from the cryptic plasmid using the kanamycin and chloramphenicol antibiotic resistance cassettes [23]. This clean background strain has been named YG5151 and used in the subsequent tests as a host for the polymerase expression system. As for control, we have used this strain transformed with the plasmid pKM101 expressing PolRI in its native form (strain YG9056) along with the standard Ames tester strains TA100 and TA1535 (Table 1).

### Spontaneous mutagenesis levels in the new strains

The strong mutagenesis promoting ability of PolRI correlates with its ability to elevate spontaneous mutation levels as can be seen in Fig. 1. The spontaneous mutant yields in strains expressing PolRI are well above the standard TA1535 background. The YG9028 strain expressing the activated PolRI has the highest spontaneous mutation level that is still marginally elevated after the *lac* promoter induction with IPTG. Due to its very high activity and affinity for the β-subunit sliding clamp [25], PolRI* promotes spontaneous mutagenesis even at basal leaky expression from the *lac* promoter and higher expression levels are actually toxic to the bacteria. We have observed strong toxicity of PolRI* expression when induced with higher IPTG concentrations added e.g. directly to the bottom agar. On the other hand the SamAB polymerase doesn’t contribute to mutagenesis to any significant extent when expressed natively but raises spontaneous mutagenesis when overexpressed from the *lac* promoter in the activated SamA’ + SamB form and upon further induction with IPTG (strain YG9029, Fig. 1). The SamA’ protein seems to counteract the PolRI* toxicity when substituted for MucA’ in strain YG9033 and the ability of SamB to support spontaneous mutagenesis is lost when its SamA’ partner is substituted with MucA’ (strain YG9032).

### Chemical mutagenesis

According to the strength of mutagenic response to the tested compounds of the used strains, three different patterns of response can be inferred from the graphs presented in Figs. 2, 3, 4, 5 and 6. First, in the case of AF-2, 1,8-DNP and AFB1 the native PolRI expressed under the SOS control from the plasmid pKM101 is better at promoting mutagenesis than its overexpressed active form PolRI*. Cisplatin can be also assigned this pattern since the overexpressed activated DNA polymerases were unable to promote its mutagenesis at all (data nor shown). Second, in the case of MMS, the overexpressed activated PolRI* promotes mutagenesis better than the native pKM101-born PolRI (Fig. 3) and third, the plasmid born Y-family DNA polymerases such as PolRI actually reduce the mutagenesis as in the case of AG (Fig. 6). The SamA’ + SamB proteins were able to support the mutagenesis in a dose dependent manner in the case of AF-2 and 1,8-DNP (Figs. 2 and 5) and the SamA’ accessory subunit was essential for this function since its substitution with MucA’ abolished the mutagenic response. The chimeric combination of SamA’ + MucB was able to promote the mutagenesis particularly by 1,8-DNP (Fig. 5) but also by AF-2, AFB1 and MMS better at higher expression levels induced by IPTG (Figs. 2, 3, 4). This contrasts with the native combination of MucA’ + MucB in PolRI* which was efficient at promoting mutagenesis only at lower expression level without adding IPTG (Figs. 2, 3, 5). The unique mutagen AG defies the common sense for the mutator DNA polymerases like PolRI as these polymerases actually suppress its mutagenesis and this effect can be even enhanced by their elevated expression (Fig. 6 and Table 2). AG was also the only mutagen able to induce reversions at the *hisG46* allele in the absence of any Y-family DNA polymerase.

## Discussion

To help better understand the activities and specificities of the episomal Y-family DNA polymerases present in the Ames tester strains we have subcloned their subunits on separate combinable plasmids. We have also replaced their SOS-based transcription, translation and posttranslation (proteolytic) regulation with the highly efficient *lac*-promoter driven system controllable by IPTG [19]. Previously, we had tested the activities of these engineered polymerases at the *hisG428* and *hisD3052* hotspots for UV-radiation and chemically induced mutagenesis in the Ames tester strains [19, 20]. To complete the target “trilogy”, we have now introduced this PolRI* and SamA’B expression system into the *hisG46* Ames test background and looked at the response to the following 5 classical mutagens: Furylfuramide (AF-2) is a member of the class of acrylamides formerly used as a food preservative in Japan and withdrawn from the market following suspicions of carcinogenicity [26]; methyl methanesulfonate (MMS) is an alkylating agent tested clinically as a cancer chemotherapeutic that acts as a strong mutagen and is reasonably anticipated to be human carcinogen [27, 28]; aflatoxin B1 (AFB1) is a potent hepatotoxic and hepatocarcinogenic mycotoxin produced by the *Aspergillus flavus* group of fungi which is mutagenic, teratogenic, and causes immunosuppression in animals [28, 29]; 1,8-dinitropyrene (1,8-DNP) is found in particulate emissions from combustion products of diesel exhaust and is anticipated to be a human carcinogen [28, 30]; azidoglycerol (AG) is a powerful non-carcinogenic mutagen genotoxic to bacteria, fungi and plants with a unique narrow point mutation specificity [31]. Additionally, we have also tested the antineoplastic agent cisplatin [32] known to form interstrand DNA crosslinks similarly to UV-light but we do not present the data in detail because it only showed some mutagenicity in the strains expressing PolRI from the plasmid pKM101 and not in our new strains expressing the activated PolRI*. 1,8-DNP and AF-2 are very powerful mutagens compared to the other agents in terms of how many revertants per plate are induced at the same molar amount of chemical according to Table 2.

Previously, we have already demonstrated, that the plasmid-born PolRI is essential for the lipid-peroxide induced mutagenesis at this GC *hisG46* and the similar AT *hisG428* base-substitution hotspots in *S. typhimurium* [23, 33]. In the current work the PolRI Y-family DNA polymerase or its SamA’B counterpart also appeared essential for producing any mutations after the exposure to AF-2, MMS, AFB1, 1,8-DNP as well as cisplatin. In striking contrary, the mutagenic action of AG has been inhibited by these Y-family DNA polymerases (Fig. 6). The mutagenicity of AG is similarly to other azides independent on the SOS-system in bacteria and produces very narrow G:C to A:T transition mutation spectrum [21, 31]. The inhibition of AG mutagenesis by PolRI could mean that the ultimate azide DNA lesion, which is still enigmatic, can be handled in an *error free* manner by the Y-family TLS polymerases. However, given the lack of SOS-induction after the exposure of bacteria to azides [21] this is unlikely to significantly contribute to the mutation yield under the standard conditions. It has been suggested that azido pyruvate could represent the ultimate DNA reactive mutagen in azide mutagenesis [34]. Given the very narrow mutation spectrum, it is conceivable that only the dNTP pool gets modified directly and a specific altered nucleoside is then incorporated into the nascent DNA strand by a DNA polymerase. In fact, we have observed the same mutation spectrum and dependence on DNA repair for the nucleoside analogue 8-azidoadenosine (preliminary data not shown) which could therefore represent suitable ultimate azide DNA lesion candidate. What is true for other standard mutagens is seen in reverse in AG mutagenesis (Fig. 6) such as the native MucA’B and SamA’B combinations are more inhibitory that the heterogenous MucA’ + SamB and SamA’ + MucB combinations. An exception is only the strain YG5151 which, after the deletion of PolV, shows decreased mutagenic response to AG suggesting possible “conventional” role of the *Salmonella* chromosomal *umuDC*_ST_ genes in azide mutagenesis. This very rare inhibitory effect of a PolV-type DNA polymerase on the induced mutagenesis can be only seen in a few rare cases such as e.g. the frameshift mutations induced by Glu-P-1 [10].

The shorter MucA’ and SamA’ subunits of the polymerases seem to be essential for the base-change type mutagenesis as the expression of the sole MucB PolRI catalytical subunit (data not shown) did not promote any mutagenesis including 1,8-DNP which has been shown to be mutagenic in the presence of MucB alone in the case of nitropyrene − 2 GC frameshift mutagenesis [20]. When highly overexpressed, upon the induction with IPTG, SamA’B has been more active in the 1,8-DNP mutagenesis than PolRI* (Fig. 5) but in the case of AF-2 and AFB1 the PolRI* seems to have the upper hand (Figs. 2 and 4). The lower activity of PolRI* expressed from the *lac*-promoter driven system compared to the native SOS-controlled operon in the case of AF-2, AFB1 and 1,8-DNP is probably due to its stoichiometric excess related to the other processive chromosomal DNA replicases competing for binding sites on the sliding clamp [25] or could be due to yet undiscovered third subunit of PolRI encoded on the pKM101 plasmid similarly to the case of the *impCAB* operon [4, 35]. The optimal level of the PolRI catalytical subunit for different DNA adducts may be linked to its occupancy at the 3 binding sites on a β-subunit sliding clamp. The sliding clamp probably acts as a tool-belt holding all polymerases needed for a complete lesion bypass together [36]. If both incorporation and extension steps during TLS across specific DNA adduct can be performed only by PolRI, the TLS would benefit from higher PolRI expression levels. However, if PolRI performs e.g. just the 3′ misaligned primer extension step, lower PolRI expression level may be optimal relative to the level of the other DNA polymerase performing the first (mis) incorporation step in order not to out-compete it from the sliding clamp platform. In fact the complete lesion bypass by a single DNA polymerase subunit can be mutagenic while the bypass by combined different DNA polymerase subunits is often *error free* such as in the case of the thymine glycol DNA adduct [37].

We have also reported previously that the mutagenesis enhancing potential of PolRI* decreases with its increased expression while it increases in the case of SamA’B. This was true in the case of UV-light induced reversions at the AT-base substitution target *his*G428 [19] as well as in the cases of AF-2, AFB1, nitropyrenes, nitrofluorene and Glu-P-1 induced − 2 GC frameshifts at the *his*D3052 target of the Ames test [20]. In our present work we are seeing the same behavior at the GC-base substitution target *his*G46 clearly in the case of AF-2 and 1,8-DNP. In contrary to the previous work, however, the MucB catalytic subunit of PolRI alone was not able to promote any mutagenesis induced by 1,8-DNP but was able to do so in the heterogenous combination with the SamA’ accessory subunit not only in the case of 1,8-DNP, but also to some extent in the case of AFB1, MMS and AF-2.

The potency of PolRI, however, seems to increase with its expression level in the case of spontaneous mutagenesis (compare Fig. 1 to [19]). The *lac*-promoter driven PolRI* was also more efficient than the SOS plasmid born PolRI in the case of MMS alkylating mutagenesis (Fig. 3) what parallels the findings of McNally et al. that higher expression level of *mucAB* is more stimulating in the MMS-induced mutagenesis [38]. We have also previously shown that “more is better” for PolRI expression in the case of crotonaldehyde induced mutagenesis in the same Ames test strain background [23]. Actually the spontaneous mutants in bacteria are likely arising from endogenous alkylating damage to DNA as has been demonstrated by knocking down its repair by the *ogt*_ST_ methyltransferase in *S. typhimurium* [39]. On the other hand in humans not only endogenous [40] but also chemically-induced cancer-driving mutations such as that at the p53 codon #249 after exposure to the hepatocellular carcinogen AFB1 arise from DNA lesions induced by lipid peroxidation products like crotonaldehyde [41]. In this respect our new strain offering the possibility to “fine-tune” the PolRI* overexpression level with IPTG can help to more faithfully evaluate potential human mutagens such as the longer chain lipid peroxides which are difficult to detect in the classical Ames test [40, 42]. Overall Ames strains expressing the activated episomal Y-family TLS DNA polymerases can be useful for the detection of certain mutagens relevant to mammalian cells because they showed increased sensitivity to the propano-adduct forming lipid peroxidation products [23] but decreased sensitivity to non-carcinogenic Ames mutagens such as the azides.

The propano-adduct forming lipid peroxidation products such as crotonaldehyde and 4-hydroxy-2-nonenal have been implicated in carcinogenesis in multiple studies [40, 43–45] and are even considered to be the principal carcinogens after the exposure to other environmental mutagens such as AFB1, urban air pollution, alcohol and cigarette smoke [41, 46, 47] or during wok cooking [48]. For instance crotonaldehyde is according to IARC considered only as a Class 3 human carcinogen [49] but it is likely the principal carcinogen after the exposure to the Class 1 human carcinogen acetaldehyde [47, 50, 51] or Class 2B human carcinogen N-nitrosopyrrolidine [52]. On the other hand the mutagen sodium azide has been shown to be non-carcinogenic in a 2-year rat study [53, 54] and its principal metabolite azidoalanine did not show any appreciable genotoxicity in mammalian cells as measured by SCEs and unscheduled DNA synthesis in Chinese hamster cells and human skin fibroblasts [55]. Likewise, AG did not enhance either chromosomal aberrations nor SCEs in human peripheral lymphocytes even at doses partially inhibiting cell division [56].

Ultimately we believe in introducing the human DNA polymerases critical for mutagenesis in mammalian cells directly into the Ames tester strains while adapting them to the prokaryotic expression and DNA replication machineries similarly as we have done in the case of the human DNA polymerase η [57]. The higher numbers of control revertant colonies in the PolRI* expressing strains could be actually advantageous for the miniaturized Ames test formats which face basal mutant yield measurement difficulties [58]. Our system also demonstrates that the increased activity of PolRI compared to other related DNA polymerases like SamAB is not merely due to its better proteolytic activation as suggested previously [5] but due to its intrinsic activity possibly related to better targeting to the DNA via an interaction with the sliding clamp [25].

## Conclusions

We have isolated the MucA’, MucB, SamA’ and SamB proteins expressed on separate plasmids in a controllable manner and introduced them into 3 sets of standard Ames tester strains detecting either base substitutions at the AT and GC hotspots or the − 2 GC frameshifts. The overexpressed PolRI* (MucA’ + MucB) was less efficient at promoting chemically induced GC base substitutions at high expression levels but not in the case of alkylating agent and spontaneously induced mutations. Therefore, the new activated DNA polymerase RI overexpressing strain YG9028 can be useful in screening for alkylating type mutagens or antimutagens. We have also revealed the unique property of the PolRI mutator polymerase to actually suppress azide mutagenesis what is relevant to the mammalian system where azides are not considered to be mutagens. The SamA’ + SamB polymerase assembly, which has not been yet characterized biochemically and is a “silent passenger” in the Ames tester strains, could also promote chemically induced mutagenesis at the *hisG46* hotspot when overexpressed.

## Data Availability

The datasets used and/or analyzed during the current study are available from the corresponding author on reasonable request.

## Abbreviations

AF-2, furylfuramide: 2-(2-Furyl)-3-(5-nitro-2-furyl) acrylamide
MMS: Methylmethane sulfonate
AFB1: Aflatoxin B1
1,8-DNP: 1,8-Dinitropyrene
AG, azidoglycerol: 3-Azido-1,2-propanediol
IPTG: Isopropyl β-D-thiogalactopyranoside
*S. typhimurium*: *Salmonella typhimurium*
TLS: Translesion DNA Synthesis
PolRI: DNA polymerase RI
PolRI*: DNA polymerase RI activated by MucA protein cleavage
MucA’: Shorter active form of MucA protein
SamA’: Shorter putative active form of SamA protein
PolV: DNA polymerase V
